# Inhibitors of Nucleotide Excision Repair Decrease UVB-Induced Mutagenesis—An In Vitro Study

**DOI:** 10.3390/ijms22041638

**Published:** 2021-02-06

**Authors:** Eszter Fidrus, Csaba Hegedűs, Eszter Anna Janka, György Paragh, Gabriella Emri, Éva Remenyik

**Affiliations:** 1 Department of Dermatology, Faculty of Medicine, University of Debrecen, 98 Nagyerdei Krt, 4032 Debrecen, Hungary; fidrus.eszter@med.unideb.hu (E.F.); hegeduscsaba88@gmail.com (C.H.); janka.eszter.a@gmail.com (E.A.J.); gemri@med.unideb.hu (G.E.); 2 Doctoral School of Health Sciences, University of Debrecen, 4032 Debrecen, Hungary; 3 Department of Dermatology, Roswell Park Comprehensive Cancer Center, 665 Elm St, Buffalo, NY 14203, USA; gyorgy.paragh@roswellpark.org; 4 Department of Cell Stress Biology, Roswell Park Comprehensive Cancer Center, 665 Elm St, Buffalo, NY 14203, USA

**Keywords:** UVB radiation, UVB mutagenesis, nucleotide excision repair (NER), cyclobutane–pyrimidine dimer (CPD) photolesion, veliparib, resveratrol, arsenic trioxide, spironolactone

## Abstract

The high incidence of skin cancers in the Caucasian population is primarily due to the accumulation of DNA damage in epidermal cells induced by chronic ultraviolet B (UVB) exposure. UVB-induced DNA photolesions, including cyclobutane–pyrimidine dimers (CPDs), promote mutations in skin cancer driver genes. In humans, CPDs are repaired by nucleotide excision repair (NER). Several commonly used and investigational medications negatively influence NER in experimental systems. Despite these molecules’ ability to decrease NER activity in vitro, the role of these drugs in enhancing skin cancer risk is unclear. In this study, we investigated four molecules (veliparib, resveratrol, spironolactone, and arsenic trioxide) with well-known NER-inhibitory potential in vitro, using UVB-irradiated CHO epithelial and HaCaT immortalized keratinocyte cell lines. Relative CPD levels, hypoxanthine phosphoribosyltransferase gene mutation frequency, cell viability, cell cycle progression, and protein expression were assessed. All four molecules significantly elevated CPD levels in the genome 24 h after UVB irradiation. However, veliparib, spironolactone, and arsenic trioxide reduced the mutagenic potential of UVB, while resveratrol did not alter UVB-induced mutation formation. UVB-induced apoptosis was enhanced by spironolactone and arsenic-trioxide treatment, while veliparib caused significantly prolonged cell cycle arrest and increased autophagy. Spironolactone also enhanced the phosphorylation level of mammalian target of rapamycin (mTOR), while arsenic trioxide modified UVB-driven mitochondrial fission. Resveratrol induced only mild changes in the cellular UVB response. Our results show that chemically inhibited NER does not result in increased mutagenic effects. Furthermore, the UVB-induced mutagenic potential can be paradoxically mitigated by NER-inhibitor molecules. We identified molecular changes in the cellular UVB response after NER-inhibitor treatment, which may compensate for the mitigated DNA repair. Our findings show that metabolic cellular response pathways are essential to consider in evaluating the skin cancer risk–modifying effects of pharmacological compounds.

## 1. Introduction

The incidence of melanoma [1,2,3,4] and nonmelanoma skin cancers [4,5] is increasing in lighter skin types and is attributed to enhanced exposure of the skin to ultraviolet-B (UVB) [6,7,8]. UVB radiation induces DNA damage in epidermal cells [9]. The most common UVB-induced DNA changes are pyrimidine–pyrimidone photoproducts (6-4PPs) and cyclobutane–pyrimidine dimers (CPDs) [9,10]. These photolesions disrupt DNA structure by forming stable bonds between two adjacent pyrimidine bases [10,11]. CPDs form up to five times more frequently after UVB radiation than 6-4PPs [12,13], and CPDs are the leading cause of UV-signature mutations, specific markers for UV-induced DNA damage [11]. Wei et al. showed that CPDs show different accumulation throughout the genome, as enrichment of UV-signature mutations on specific genetic locations (mutational hotspots) can be detected [14,15].

In human cells, UVB-induced photolesions are repaired by nucleotide excision repair (NER) [16]. NER is highly effective in the repair of 6-4PPs, but less effective in repairing CPDs. Nakagawa et al. showed that 6 h after UVB irradiation, 6-4PPs are completely removed [17], while more than 40% of UVB-induced CPDs are left unrepaired even 24 h after UVB injury [17]. Furthermore, mutation data from epidermal cancer suggest that UVB-induced molecular changes are mainly attributed to CPDs [9,10,18]. Defects in the repair process cause rare genetic disorders, including xeroderma pigmentosum (XP), Cockayne syndrome (CS), and trichothiodystrophy (TTH). Patients with these diseases are extremely sensitive to sunlight and, in the case of XP, the risk of skin cancer at an early age is very high [19].

NER function can be both positively and negatively influenced by various chemical agents. Nicotinamide [20,21] and some plant derivatives [22,23,24,25] were shown to enhance NER activity in vitro. However, some chemical agents impair DNA repair [26,27,28,29,30,31,32], raising the possibility of enhanced skin cancer risk in exposed individuals. Among the repair-inhibiting drugs, veliparib, an inhibitor of poly [ADP-ribose] polymerase 1 (PARP1), is currently in clinical trials to target different malignancies [33,34,35,36]. Spironolactone [37,38] and arsenic trioxide [39,40,41] are used in clinical practice for their diuretic and chemotherapeutic properties, respectively. Resveratrol, a natural phytophenol, is a promising compound in UV protection via its anti-inflammatory [42,43,44,45], anti-oxidant [45,46], and anti-carcinogenic [47,48,49] effects. Although these chemicals are widely used, there are no in vivo data examining the risk of UVB-induced tumorigenesis in treated individuals or the role of NER inhibition in this risk. NER functionality may have no linear and obligate relationship with UVB-driven mutagenesis; other factors may also influence photocarcinogenesis.

In this study, we aimed to investigate the mutagenic effects of four chemical agents—veliparib, resveratrol, spironolactone, and arsenic trioxide—with known in vitro NER-inhibitory properties [26,29,31,50]. Since the ability of these molecules to impair NER function is well proven, our aim was to assess whether decreased repair function and increased CPD accumulation by the treatment of the tested chemicals lead to enhanced mutagenesis of epithelial cells in vitro. In addition to their impact on CPD formation and UV-induced mutation burden, we identified other molecular pathways (apoptosis, cell cycle progression, or autophagy) modified by these molecules, which have significant role in cellular UVB response.

## 2. Results

### 2.1. All Tested Chemicals Enhance CPD Formation after UVB Irradiation

To verify the NER-inhibitory effect of veliparib, resveratrol, arsenic trioxide, and spironolactone, CHO cells were pretreated with the chemicals and irradiated with 20 mJ/cm^2^ UVB. In our previous study, we evaluated the kinetics of CPD removal after UVB irradiation, and we found that most of the UV-induced lesions (~60%) are eliminated from the DNA in the first 24 h [51]. According to this and other studies aiming to assess repair efficacy [17,52], we chose to measure the relative CPD content of the cells 24 h after UVB.

**Figure 1:**
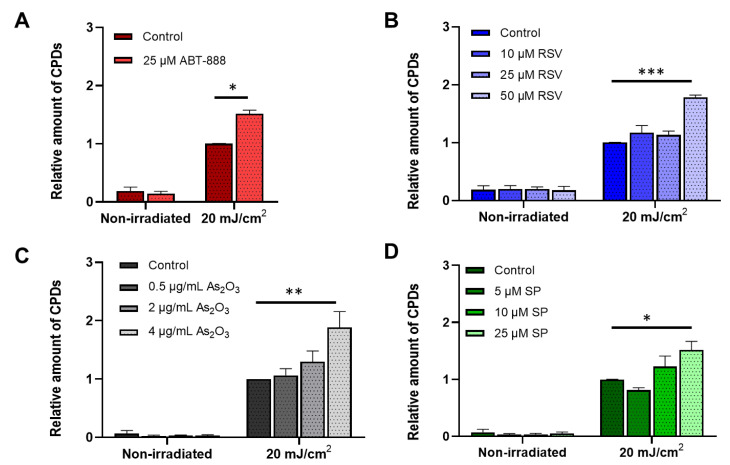
Relative number of cyclobutane–pyrimidine dimer (CPD) photolesions after ultraviolet B (UVB) irradiation. CHO cells were pretreated with (**A**) veliparib (ABT-888), (**B**) resveratrol (RSV), (**C**) arsenic trioxide (As_2_O_3_), or (**D**) spironolactone (SP), and then irradiated with 20 mJ/cm^2^ UVB. CPD lesions were detected by CPD-specific ELISA 24 h after the irradiation. CPD amounts were normalized to UVB-irradiated vehicle controls. We present the mean ± SEM of at least three independent experiments. *, **, and *** indicate statistically significant difference at *p* < 0.05, *p* < 0.01, and *p* < 0.001.

Twenty-four hours post-UVB, a large number of CPDs remained in the cellular DNA reflecting the slow repair of CPD lesions by NER [17]. The relative amount of CPDs was significantly higher in the treated groups compared to that in the nontreated counterparts. In many cases, the number of CPD lesions showed more than a 50% increase, e.g., the detectable CPD amount was 88% higher after 4 µg/mL arsenic-trioxide treatment compared with that in the sham-treated group (Figure 1). These findings are consistent with other studies showing that these molecules impair the removal of CPDs [26,28,29,31,32,50]. To validate these data, we repeated the experiments in HaCaT human keratinocyte cell line using the most effective concentrations. In our previous study, we have already presented that veliparib treatment reduces CPD repair in this cell line [29], and we also found similar results by resveratrol, spironolactone, and arsenic-trioxide treatment, too (Figure S1).

### 2.2. CPD Accumulation and UVB-Induced Mutagenesis Show a Nonlinear Relationship

**Figure 2:**
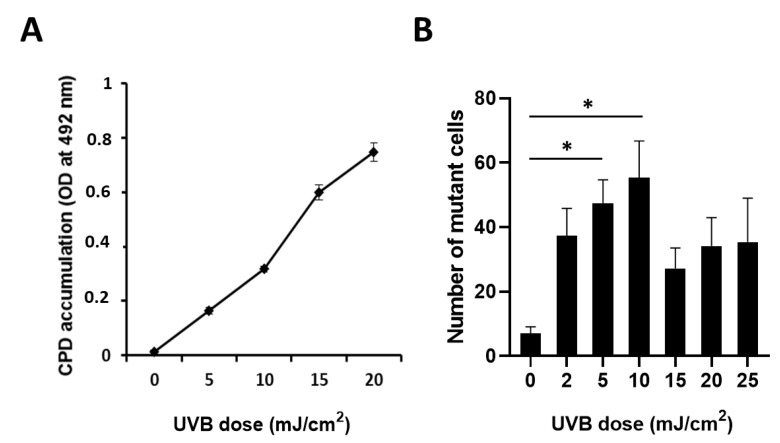
CPD accumulation and UVB-induced mutagenesis following different doses of UVB exposure. (**A**) Accumulation of CPD photolesions with increasing doses of UVB radiation in CHO cells. CPDs were detected 24 h after UVB exposure. Mean ± SEM (*n* = 3). (**B**) Number of mutated cells in response to increasing doses of UVB radiation in CHO cells after a 10-day culture in selective medium containing 6-thioguanine. Mean ± SEM, *n* = 6. * indicates statistically significant difference at *p* < 0.05.

To evaluate the mutagenic effect of UVB radiation, *HPRT* gene mutation assays were performed [53]. This assay detects cells carrying heritable mutations in the *HPRT* gene. First, we assessed the dose dependence of CPD accumulation. Our results show that CPD levels, detected 24 h after UVB irradiation, increased linearly with the UVB dose (Figure 2A) [54]. Interestingly, UVB-induced mutagenesis did not exhibit a linear dose–response relationship with CPD accumulation. UVB-induced *HPRT* mutation frequency increased from 0 to 10 mJ/cm^2^ UVB, then the mutational rate dropped at 15 mJ/cm^2^ (Figure 2B), suggesting that UVB doses with lower cytotoxic effects are more mutagenic [55]. Based on this observation, we chose 10 mJ/cm^2^ UVB in our experimental system for *HPRT* mutation detection. Figure S2 presents the linear reduction of viability after increasing UVB doses.

### 2.3. Veliparib, Arsenic-Trioxide, and Spironolactone Treatments Prevent UVB-Induced Mutagenesis

**Figure 3:**
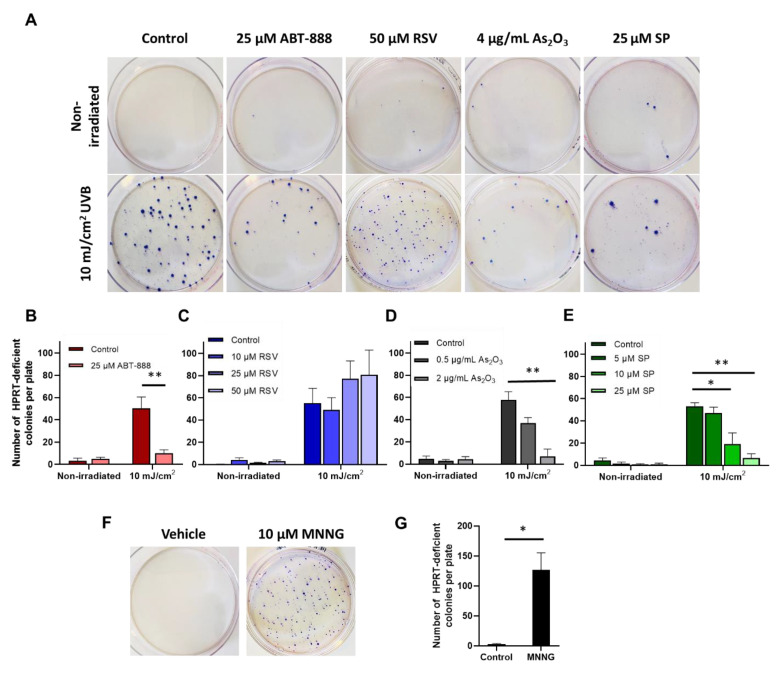
Effects of nucleotide excision repair (NER) inhibitors on UVB-induced HPRT gene mutation burden. (**A**) HPRT gene mutation assay after UVB and NER-inhibitor treatments. Cells were pretreated and exposed to 10 mJ/cm^2^ UVB radiation. HPRT mutant cells were selected in Dulbecco’s modified Eagle media (DMEM) containing 5 μM 6-thioguanine. One representative experiment from at least three independent measurements is presented. (**B**–**E**) Mean ± SEM of ≥3 HPRT gene mutation assays after treatment with (**B**) veliparib (ABT-888), (**C**) resveratrol (RSV), (**D**) arsenic trioxide (As_2_O_3_), and (**E**) spironolactone (SP). (**F**,**G**) 10 μM 1-methyl-3-nitro-1-nitrosoguanidine (MNNG) was used as a positive control for the assays. * and ** indicate statistically significant differences at *p* < 0.05 and *p* < 0.01.

To assess the effects of chemically induced NER inhibition on UVB mutagenesis (Figure 3), we examined whether the compounds increased the mutation frequency of the HPRT gene after UVB irradiation. Since HaCaT cells were extremely intolerant to 6-thioguanine (6-TG) selection medium and formed a very low number of colonies after UVB radiation, HPRT assay was only carried out on CHO cells, which is the cell line conventionally used for measuring HPRT mutagenesis [53]. Contrary to expectations, we found that veliparib, arsenic-trioxide, and spironolactone treatment decreased the number of cells carrying nonfunctional mutations in their HPRT gene after UVB exposure (Figure 3A,B,D,E). Higher concentrations of the treatment compounds decreased the mutation rates almost to baseline, which was surprising in light of the increased CPD content with the same agents 24 h after the UVB exposure. The fourth molecule, resveratrol, caused nonsignificant increases in UVB-induced mutagenesis (Figure 3C).

### 2.4. Resveratrol, Arsenic Trioxide, and Spironolactone Enhanced UVB-Induced Apoptosis

While chemically induced attenuation of NER was triggered by the test compounds, some of them also showed marked anti-mutagenic activity. Therefore, we aimed to investigate other mechanisms that can affect UVB-induced mutations. Arsenic derivatives have well-known cytotoxic effects [56,57], especially in combination with other mutagens, such as UVB [58]. Apoptosis serves as a protective mechanism to diminish mutant clone formation [59]. Therefore, we hypothesized that the applied chemicals may induce an elevated photosensitive response in the UV-exposed cells, thereby inducing the clearance of cells with unrepaired DNA damage, resulting in decreased UVB-induced mutagenesis.

Because 10 mJ/cm^2^ UVB (used for HPRT mutagenesis assay) caused a very moderate decrease in cell viability (more than 80% of the cells left viable) (Figure S2), we decided to choose 20 mJ/cm^2^ for apoptosis measurements. To verify that the UVB dose does not influence the effects of a chemical treatment on HPRT mutagenesis, we repeated HPRT mutation assays with two of the most anti-mutagenic treatments, 25 µM ABT-888 and 25 µM SP. Although 20 mJ/cm^2^ was less mutagenic than 10 mJ/cm^2^, the direction of the changes after inhibitor treatments remained the same (Figure S3).

**Figure 4:**
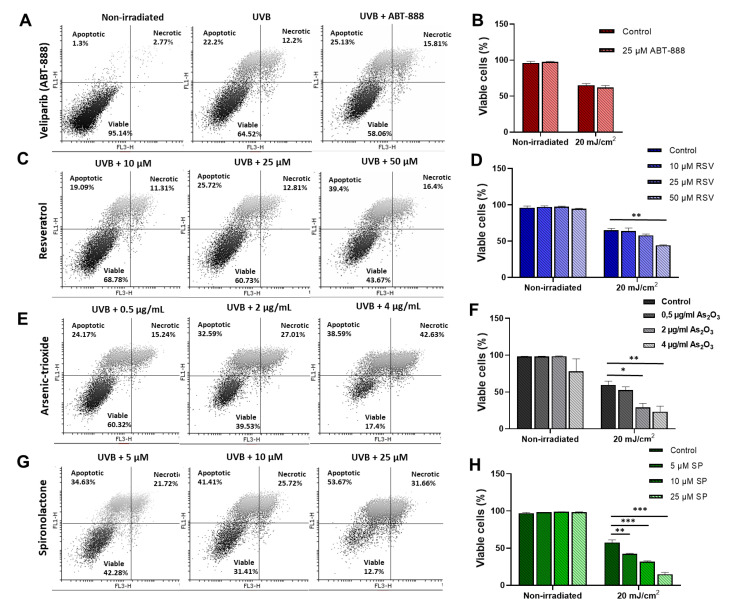
Changes in UVB-induced alterations in cell viability induced by pretreatment with NER-inhibitory molecules. Cells were pretreated with the indicated concentrations of (**A**,**B**) veliparib (ABT-888), (**C**,**D**) resveratrol (RSV), (**E**,**F**) arsenic trioxide (As_2_O_3_), or (**G**,**F**) spironolactone (SP) and exposed to 20 mJ/cm^2^ UVB or left unexposed (nonirradiated). After 48 h of irradiation, apoptotic (Annexin V+/propidium iodide (PI−), necrotic (Annexin V+/PI+), and viable (Annexin V−/PI−) cells were detected by flow cytometry. (**B**,**D**,**F**,**H**) Bars represent the percentage of living cells 48 h after UVB exposure after inhibitor treatments. We calculated the mean ± SEM of three independent experiments, where *, **, and *** denote statistically significant differences at *p* < 0.05, *p* < 0.01, and *p* < 0.001.

CHO cells were pretreated with the drugs and exposed to UVB. Subsequently, i.e., 48 h after irradiation, cells were stained with Alexa Fluor 488–conjugated Annexin V (AV) and propidium iodide (PI). Viable, apoptotic, and necrotic subpopulations were determined by flow cytometry. Both arsenic-trioxide and spironolactone treatments increased the proportion of dead cells in response to UVB. At higher treatment concentrations (4 μg/mL arsenic trioxide or 25 μM spironolactone), the mean percentage of living cells was between 11% and 23%. In the group exposed to UVB only, more than 50% of the cells were viable 48 h after the irradiation (Figure 4E–H). Resveratrol, the only tested molecule without detectable anti-mutagenic properties, induced only a mild increase in UV-induced apoptosis at high concentrations (Figure 4C,D); this is probably one of the reasons for the unaltered mutagenic response. Surprisingly, veliparib, a molecule with high DNA-repair-inhibitory properties and anti-mutagenic effects, caused no alterations in cell viability after UVB (Figure 4A,B). In HaCaT cells, we observed very similar alterations by the treatments (Figure S4); however, veliparib caused a mild decrease in cell viability in our previous study [29].

### 2.5. Veliparib and Resveratrol Augment UVB-Induced Cell Cycle Arrest

**Figure 5:**
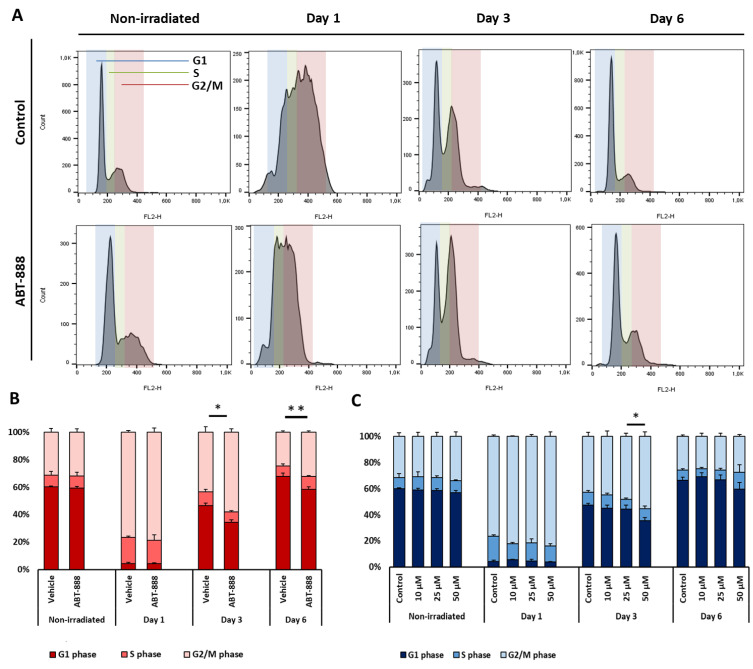
Cell cycle progression after veliparib and resveratrol treatment followed by UVB irradiation. (**A**) Cells were treated with 25 μM veliparib (ABT-888) and exposed to 20 mJ/cm^2^ UVB or left unexposed (nonirradiated). Cell cycle progression was analyzed 1, 3, and 6 days after exposure using propidium-iodide staining followed by flow cytometry. G1 (blue), S (green), and G2/M (red) phase cell populations were distinguished, as indicated. (**B**) The distribution of cells in different phases was calculated after veliparib and (**C**) different doses of resveratrol treatment (mean ± SEM; *n* = 3). * and ** denote statistically significant differences at *p* < 0.05 and *p* < 0.01, respectively.

Besides apoptosis, cell cycle arrest is one of the main cellular mechanisms that can attenuate the long-term effects of mutagenic exposure. During this process, cell division is halted in cells with unrepaired DNA lesions, extending the time for repair [60,61]. To assess whether the tested molecules can modify UVB-induced cell cycle arrest, we analyzed cell cycle progression of CHO cells 1, 3, and 6 days after 20 mJ/cm^2^ UVB exposure. One day post-UVB, a large number of cells was detected in the G_2_/M phases in every UV-irradiated group, consistent with previous findings showing that UVB-radiation-induced cell cycle arrest is mainly manifested at G_2_/M [62]. Restoration of the cell cycle began at 3 days after UVB exposure and was nearly indistinguishable from the nonirradiated group 6 days after UVB radiation. When cells were pretreated with 25 μM veliparib, the percentage of cells in the G_2_/M phase showed a mild but statistically significant increase compared to that in the vehicle control. The increase lasted up to 6 days after the exposure (Figure 5A,B), suggesting that veliparib treatment extends the recovery time of cells from DNA damage, which possibly contributes to its anti-mutagenic effect. The increase of UVB-induced cell cycle block and decreased proliferative capacity after veliparib treatment were also observed in HaCaT cells [29]. Resveratrol also caused a moderate elevation in the G_2_/M block 3 days after the UVB, but this difference was only short-term and was not detectable 6 days after UVB exposure (Figure 5C). HaCaT cells did not show change in cell cycle progression after resveratrol treatment (Figure S5). Arsenic-trioxide and spironolactone treatments did not affect cell cycle progression after UVB either in CHO (Figure S6A,B) or in HaCaT cells (Figure S6C,D).

### 2.6. Altered Protein Expression in Diverse Stress–Response Pathways May Orchestrate UVB-Induced Mutagenesis

**Figure 6:**
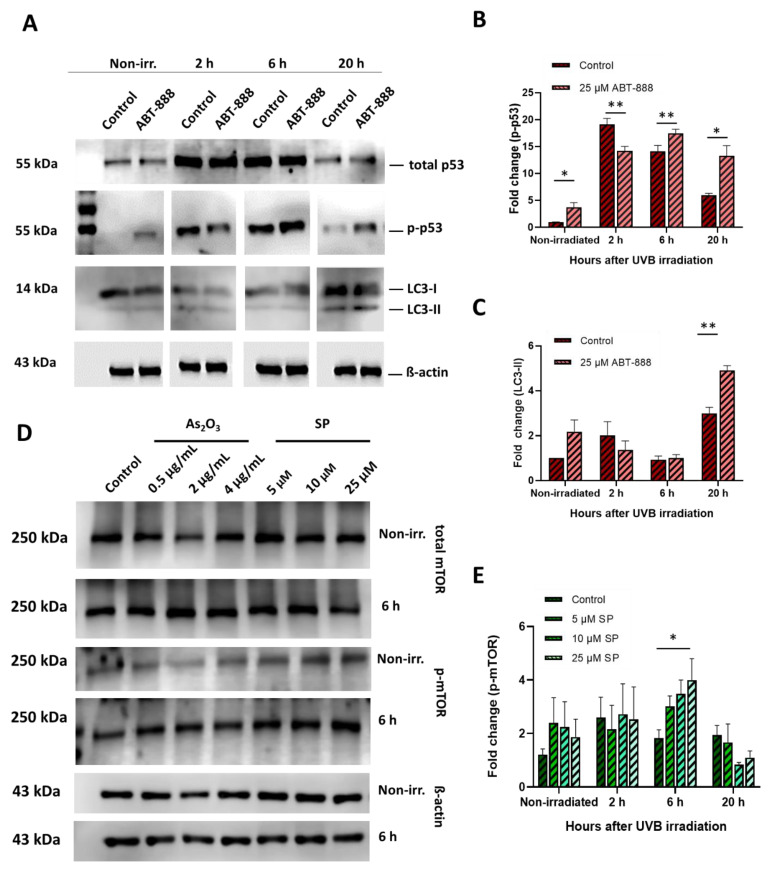
Protein expression and activation changes after veliparib (ABT-888), arsenic-trioxide (As_2_O_3_), or spironolactone (SP) treatment followed by UVB irradiation. Cells were treated with 25 μM veliparib (ABT-888) or different concentrations of As_2_O_3_ or SP and exposed to 20 mJ/cm^2^ UVB or left unexposed (nonirradiated). Control cells were UVB irradiated without any additional treatment. (**A**) Level of total tumor protein 53 (p53), phospho-p53 (p-p53) and microtubule-associated protein 1A/1B-light chain 3 protein (LC3-I and LC3-II) following ABT-888 treatment and (**D**) total mammalian target of rapamycin (mTOR) and phosphorylated mTOR (p-mTOR) (following SP or As_2_O_3_ treatment) were detected by Western blotting. Bars represent the mean of (**B**) *n* = 6 for p53 phosphorylation; (**C**) *n* = 3 for LC3-II expression after ABT-888 treatment and (**E**) *n* = 4 for mTOR phosphorylation after SP or As_2_O_3_ treatment. Data were normalized to ß-actin. * and ** denote statistically significant differences at *p* < 0.05 and *p* < 0.01, respectively.

We aimed to identify general upstream regulators at the protein level that can shed light on the anti-mutagenic nature of the compounds. Thus, we looked for changes in the expression or activation levels of proteins involved in key pathways linked to DNA damage with UVB-induced mutagenesis. First, we measured the phosphorylation level of p53 protein and the expression of the phosphatidylethanolamine conjugated form of microtubule-associated protein 1A/1B-light chain 3 protein (LC3-I-II). Phospho-p53 and LC3-I and II are widely accepted markers of DNA damage sensing and cellular autophagy, respectively [63,64]. Western blot analysis revealed that LC3-II expression was increased after UVB exposure, and veliparib treatment enhanced these effects 20 h post-UVB (Figure 6A,B). Thus, PARP1 inhibition by veliparib promotes UVB-induced autophagy [29]. Similar to LC3 expression, p53 phosphorylation levels increased after UVB irradiation and this effect was augmented by PARP inhibition (Figure 6A,C). Total p53 expression was also enhanced by UVB exposure, but veliparib treatment did not affect it (Figure 6A). No differences in LC3 expression (Figure S7A–C) or p53 phosphorylation levels (Figure S7D–F) were detected after resveratrol, spironolactone, or arsenic-trioxide treatments, suggesting that the observed cellular changes were mediated by p53-independent mechanisms in these cases.

In addition to the p53 pathway, activation of the mammalian target of rapamycin (mTOR) signaling is another key mechanism for the regulation of diverse stress–response pathways, including apoptosis, senescence, and autophagy. In contrast to p53, phosphorylated mTOR promotes the survival and proliferation of UVB-exposed cells [65]. We found, that arsenic-trioxide and spironolactone treatments increased mTOR phosphorylation 6 h after UVB, but this increase was only statistically significant after spironolactone treatment. Total mTOR expression was not affected (Figure 6D,E). The other two chemicals did not affect mTOR phosphorylation after UVB (Figure S8A–C).

## 3. Discussion

Gross NER defects in XP patients result in accelerated photocarcinogenesis. Several human medications are effective inhibitors of NER [26,28,29,31], raising the possibility that the clinical application of these molecules enhances photocarcinogenesis. Although some of these molecules are widely used in clinical practice, in vivo therapeutic application of these compounds is not associated with increased risk of photocarcinogenesis [35,66,67].

We confirmed that all these test molecules impair the elimination of CPD photolesions from DNA after UVB irradiation. Three molecules—veliparib, spironolactone, and arsenic trioxide—exerted strong anti-mutagenic effects (*HPRT* gene mutation assay), while resveratrol not affected UVB-induced mutation formation. Spironolactone and arsenic trioxide induced a marked loss in cell viability upon UVB exposure, while inhibition of PARP1 by veliparib caused a prolongation of UVB-induced cell cycle arrest.

The detected anti-mutagenic effects of the tested compounds could be interesting in the aspect of synthetic lethality, which is a promising therapeutical approach for the treatment of various cancers [68]. Inhibitors of PARP1 are already investigated due to their synthetic lethal effects with the combination of pre-existing *BRCA1/2* (Breast Cancer gene 1/2) loss-of-function mutations [69,70]. Our findings that these inhibitors are able to reduce the survival of cells carrying mutations, also support the hypothesis that selective elimination of cancerous cells can be achieved by inducing defects in cellular repair pathways, supplementing the deleterious effects of other genetic deficiencies. The possibility of testing the other anti-mutagenic chemicals (besides veliparib) as synthetic lethal compounds should be also considered—especially in the case of spironolactone, which has markedly less harmful side effects compared to arsenic trioxide.

These results suggest, that possible synthetic lethal properties of the chemicals can originate from their ability to inhibit specific subunits of the NER complex. Although PARP1 is involved in the recognition of CPDs and thus in the initiation of the NER process through the activation of DDB2 (DNA damage-binding protein 2) [71,72] and XPC (Xeroderma pigmentosum, complementation group C) [73], this multi-faceted protein also plays a role in the regulation of other repair pathways. For example, PARP1 has been shown to interact with CSB (Cockayne syndrome group B), a protein involved in both transcription-coupled NER and base excision repair (BER) [71,74]. Thus, its synthetic lethality is may be linked to a complex dysregulation of DNA repair. In contrast, spironolactone was found to inhibit NER by inducing the rapid proteosomal degradation of the XPB (Xeroderma pigmentosum, complementation group B) subunit [31,32], which suggests a more specific interaction between pre-existing mutations and defective NER repair resulting in the elimination of genetically damaged cells. Arsenic trioxide was also shown to inhibit the XPC subunit [28,50], but this molecule has other versatile effects. For instance, arsenic trioxide regulates the survival of damaged cells through the induction of Bax/caspase-3 pathway [57,75], ROS (relative oxygen species) production [57], and the downregulation of survivin [75]. Thus, its lethal effects on UV-exposed cells cannot be merely explained by XPC inhibition. The underlying mechanism of resveratrol-induced defect in CPD removal is less known, but Keuser et al. found a significant compaction of the chromatin structure after resveratrol treatment. Resveratrol induces the activation of sirtuins (SIRTs), which are members of the class III histone deacetylases (HDACs), and regulates various cellular pathways including DNA damage recognition and repair through the deacetylation of target proteins. Furthermore, deacetylation-mediated chromosome remodeling via SIRTs may influence the accessibility of repair proteins to the damaged DNA and result in the accumulation of CPDs and other types of DNA lesions, as well [26].

P53 is considered to be the guardian of the genome; it regulates the balance between pro- and anti-apoptotic signals during the stress response [64]. In our study, veliparib significantly enhanced UV-induced p53 phosphorylation. This is intriguing in the view of other studies, which found that the application of PARP1 inhibitors in cancer therapy is more effective in p53-deficient cells [76,77]. This is a result of direct interaction between PARP1 and p53 protein, where the status of p53 (gain-of function/loss-of-function) dictates the outcome of cell survival. For example, loss-of-function mutations of p53 were associated with higher sensitivity to PARPi (inhibitor of poly-(adenosine diphosphate [ADP]) ribose polymerase) [78,79,80]. These results suggest that increased activation or gain-of-function mutation of p53 following chemically induced PARP1 inhibition may serve to protect cells from intensive genome instability due to the loss of PARP1 function.

Except veliparib, none of the other molecules caused significant alterations in p53 phosphorylation, suggesting that changes are at least partly mediated by p53-independent pathways in these cases. The phosphorylation of mTOR, involved in cell survival, was increased by spironolactone, in contradiction to the strong apoptotic response. We hypothesize that increased mTOR phosphorylation is a cellular strategy to counteract the cytotoxic effect of SP.

Autophagy represents another key strategy in cell survival [64], which was enhanced by ABT-888 treatment. However, the role of autophagy in tumorigenesis is quite controversial. While autophagy helps remove damaged cellular organelles and thereby prevents tumor formation, it can also fuel metabolism by recycling damaged molecules to promote the survival of pre-cancerous cells during metabolic stress in a nutrient-deficient environment [81].

We observed that the mutagenic effect of UVB and CPD accumulation did not exhibit parallel increases, as UVB-induced mutagenesis decreased at higher UVB doses. This shows that there is no obligate linear relationship between repair activity and UVB mutagenesis, but other factors should be also considered while assessing the possible risk of skin cancer induction by a chemical treatment. Alterations in the cellular UVB response, such as elevated apoptosis, cell cycle arrest, and autophagy, may play a significant role in counterbalancing the negative effect of repair inhibition on mutagenesis.

In conclusion, we demonstrated that UVB-induced mutagenesis is a highly complex process even in an in vitro model, and decreased cellular repair activity does not necessarily result in elevated mutagenicity. These results suggest that, if a molecule inhibits DNA repair in vitro, its effects on other cellular processes also need to be assessed before its mutagenic potential can be predicted. Furthermore, DNA repair inhibitors need not be considered necessarily mutagenic. We also showed that three out of four compounds reduced UVB-induced mutagenesis in vitro, which suggest that further in vivo studies are warranted to establish the safety of these molecules.

## 4. Materials and Methods

### 4.1. Cell Culture

CHO-K1 (Chinese hamster ovary; ATCC, Manassas, VA, USA) and HaCaT (immortalized human keratinocyte; ATCC, Manassas, VA, USA) cells were cultured in 4500 mg/L glucose containing Dulbecco’s modified Eagle media (DMEM, Biosera, Budapest, Hungary) supplemented with L-glutamine (Biosera, Budapest, Hungary), 10% heat-inactivated fetal bovine serum (FBS; Biosera, Budapest, Hungary), and 0.5% antibiotic/antimycotic solution (Biosera, Budapest, Hungary). Cells were maintained in a humidified incubator at 37 °C with 5% CO_2_ atmosphere.

### 4.2. Cell Treatment

Cells were harvested with trypsin-EDTA (Biosera, Budapest, Hungary) and then seeded in six-well plates for the hypoxanthine phosphoribosyltransferase (*HPRT*) gene mutation assay or 24-well plates for all other experiments. At ~80% confluence, cells were pretreated with 25 μM ABT-888 (PARP1 inhibitor, veliparib, Selleckchem, Houston, TX, USA), 10–50 μM resveratrol (Abcam, Cambridge, UK), 5–25 μM spironolactone (Selleckchem, Houston, TX, USA), or 0.5–4 μg/mL As_2_O_3_ (Sigma-Aldrich, St. Louis, MO, USA) solution. In the case of veliparib treatment, we chose the concentration that caused marked inhibition of PARP1 protein, according to our previous [29] and current experiments (Figure S9). For the other chemicals, we identified three different concentrations due to their more complex and not fully understood mode of action—based on prior published data [26,27,30,31,32]. As_2_O_3_ was dissolved in 1 M NaOH (Sigma-Aldrich, St. Louis, MO, USA) and diluted in Dulbecco’s phosphate-buffered saline (DPBS; Biosera, Budapest, Hungary). Other chemicals were dissolved in dimethyl sulfoxide (DMSO, Sigma-Aldrich, St. Louis, MO, USA). Pretreated cells were incubated for 120 min at 37 °C before UVB irradiation.

### 4.3. UVB Irradiation

After pretreatment and incubation, the culture medium was removed, and cells were covered with a thin layer of DPBS. Cells were irradiated with 20 mJ/cm^2^ UVB, using two UVB broadband tubes (TL-20W/12 RS; Philips, Eindhoven, The Netherlands). For the *HPRT* gene mutation assay, the UVB dose was reduced to 10 mJ/cm^2^, as it was found to be the most mutagenic for CHO cells (Figure 2). The proper dosage of UVB was determined by a UVX Digital Radiometer (UVP Inc., San Gabriel, CA, USA). After irradiation, the DPBS was replaced by DMEM supplemented as described above.

### 4.4. CPD-Specific Enzyme-Linked Immunosorbent Assay (ELISA)

A CPD-specific ELISA was performed as previously described by Boros et al. [82]. Genomic DNA was extracted by an Invitrogen™ PureLink™ Genomic DNA Mini Kit (Thermo Fisher Scientific, Waltham, MA, USA) 24 h after the UVA irradiation. Flat-bottomed 96-well plates were coated with 0.003% protamine-sulfate and incubated at 37 °C to completely dry. DNA was denatured at 100 °C for 10 min, then immediately chilled on ice for 15 min. Denatured DNA was distributed to wells in triplicate (15 ng DNA to each well) and incubated at 37 °C overnight. Plates were washed with PBS (Biosera, Budapest, Hungary) containing 0.05% Tween-20 (Amresco, Solon, OH, USA) (PBS-T) and incubated with 150 μL/well 5% FBS at 37 °C for 30 min to prevent nonspecific antibody binding. After washing plates three times with PBS-T, anti-CPD monoclonal antibody (clone TDM-2, dilution 1:1500, Cosmo Bio Co., Ltd., Tokyo, Japan) was added to each well. Plates were incubated at 37 °C for 60 min. After washing three times, HRP-conjugated anti-mouse IgG secondary antibody (dilution 1:3000, Bio-Rad, Hercules, CA, USA) was added and plates were incubated at 37 °C for 30 min. Plates were washed three times with PBS-T and once with 150 μL/well citrate-phosphate buffer (0.51% C_6_H_8_O_7_.H_2_O (Sigma-Aldrich, St. Louis, MO, USA) and 0.73% Na_2_HPO_4_ (Sigma-Aldrich, St. Louis, MO, USA) in distilled water; pH 5.0). Substrate solution (0.04% o-phenylenediamine (Sigma-Aldrich, St. Louis, MO, USA) and 0.006% H_2_O_2_ (Sigma-Aldrich, St. Louis, MO, USA) dissolved in citrate-phosphate buffer with H_2_O_2_ added to the solution, when o-phenylenediamine was completely dissolved) was added to each well. Plates were incubated until the appropriate color intensity appeared. To stop the enzyme reaction, 50 μL/well 2 N H_2_SO_4_ (Sigma-Aldrich, St. Louis, MO, USA) was added to each well. Absorbance was measured at 492 nm using an Epoch Microplate Spectrophotometer (BioTek, Budapest, Hungary).

### 4.5. HPRT Gene Mutation Assay

CHO cells were cultured in DMEM containing HAT (hypoxanthine–aminopterin–thymidine; HAT Media Supplement (50×) Hybri-Max™; Sigma-Aldrich, St. Louis, MO, USA) for a week to eliminate preexisting HPRT-mutant cells from the culture. CHO cells were treated with the previously specified inhibitor molecules and exposed to 0–25 mJ/cm^2^ UVB. Cells were cultured for one more week and then harvested with trypsin-EDTA (Biosera, Budapest, Hungary). In the case of each sample, an equal number of cells (1 × 10^6^) were seeded into 100 mm Petri dishes in selective DMEM containing 5 μM 6-thioguanine (6-TG; Sigma-Aldrich, St. Louis, MO, USA). The 6-TG-resistant cells were allowed to form visible clones for 10 days. Clones were washed with PBS, fixed with 100% methanol (Sigma-Aldrich, St. Louis, MO, USA) for 10 min, and stained with May–Grünwald–Giemsa (Molar Chemicals, Halásztelek, HU, Hungary). HPRT-mutant colonies were counted. For the positive control, 10 μM 1-methyl-3-nitro-1-nitrosoguanidine (MNNG; TCI Europe N.V., Zwijndrecht, Belgium) was used.

### 4.6. Apoptosis Assay

Cell viability was measured 48 h after UVB irradiation using Alexa Fluor 488–conjugated Annexin V/propidium iodide (PI) dual staining (apoptosis assay, Alexa Fluor^TM^ 488 Annexin V/Dead Cell Apoptosis Kit, Thermo Fisher Scientific, Waltham, MA, USA). The supernatant of the cells was collected, living cells were harvested with trypsin-EDTA and added to the supernatant. To avoid the loss of apoptotic cells, cell culture media was not changed between UVB exposure and viability measurement. Cells were labeled according to the manufacturer’s instructions. Cells were analyzed by flow cytometry using a FACS Calibur (Becton Dickinson, San Jose, CA, USA) flow cytometer and CellQuestPro software 5.2 (Becton Dickinson, San Jose, CA, USA). Fluorescence intensity was measured in the FL-1 (for Annexin V) and FL-3 (for PI) channels. For data evaluation, FlowJo 10.6.2. (Becton Dickinson, San Jose, CA, USA) flow cytometry software was used.

### 4.7. Cell Cycle Analysis

Cell cycle progression was quantified 1, 3, and 6 days after UVB irradiation. Cells were harvested with trypsin-EDTA, washed with DPBS, and fixed with ice-cold 80% ethanol (VWR, Radnor, PA, USA). Equal numbers of cells were centrifuged at 3500 rpm, for 5 min and re-suspended in 50 μL DPBS containing 0.2 mg/mL RNase A (Sigma-Aldrich, St. Louis, MO, USA), 0.1 μL Triton-X 100 (Amresco, Solon, OH, USA), and 5 mg/mL PI (Thermo Fisher Scientific, Waltham, MA, USA). Samples were incubated at 37 °C for 45 min and then supplemented with 0.5% bovine serum albumin (BSA; VWR, Radnor, PA, USA). Cell cycle progression was analyzed by flow cytometry with an FACS Calibur and fluorescence was measured on the *x*-axis in the FL2-A channel. Doublet discrimination was performed for single-cell analysis. FlowJo software was used for analyzing the data.

### 4.8. Western Blot

Cells were lysed in RIPA (Radioimmunoprecipitation assay) buffer containing protease-inhibitor cocktail (dilution 1:1000) 2, 6, or 24 h after UVB irradiation. Lysates were centrifuged at 15,000 rpm for 5 min at 4 °C. Protein concentration in the supernatants was measured using a Pierce BCA (Bicinchoninic acid) assay kit (Thermo Fisher Scientific, Waltham, MA, USA). Protein samples were mixed with 5× loading buffer (bromophenol blue (0.25%), β-mercaptoethanol (5%; Sigma-Aldrich, St. Louis, MO, USA), glycerol (50%; Sigma-Aldrich, St. Louis, MO, USA), SDS (sodium dodecyl sulfate; 10%; Duchefa Biochemie, Haarlem, The Neatherlands), Tris-HCl (0.25 M, pH 6.8; Sigma-Aldrich, St. Louis, MO, USA)), then boiled at 100 °C for 10 min. Proteins were separated on 7.5%, 10%, or 12.5% polyacrylamide gels, then transferred to nitrocellulose membranes (Bio-Rad, Hercules, CA, USA). Membranes were washed in TBS-T (TBS buffer containing 0.05% Tween-20), blocked in 5% nonfat dry milk for 1 h, and incubated with the primary antibody overnight at 4 °C. Antibodies used for Western blotting are listed in Supplementary Table S1. Antibodies were diluted in TBS-T containing 5% BSA. After washing with TBS-T, membranes were incubated with horseradish peroxidase (HRP)–conjugated goat anti-mouse/anti-rabbit IgG secondary antibodies (Bio-Rad, Hercules, CA, USA; dilution 1:2000) for 1 h with gentle shaking. After washing, protein bands were visualized using Pierce™ ECL Western Blotting Substrate (Thermo Fisher Scientific, Waltham, MA, USA) or SuperSignal West Femto Maximum Sensitivity Substrate (Thermo Fisher Scientific, Waltham, MA, USA). For band quantification, ImageJ 1.8.0 software (Research Services Branch, National Institute of Mental Health, Bethesda, MD, USA) was used.

### 4.9. Statistical Analysis

The normality of the population was determined using the Shapiro–Wilk test. If two groups were compared, we used independent t-test (two tailed), as the Shapiro–Wilk test showed normal distribution. When we compared three or more groups, one-way ANOVA complemented by Dunnett’s post-hoc test was used, if the data showed normal distribution. Kruskal–Wallis test complemented with Dunn’s post hoc test was performed, if the distribution of the data was not normal. Statistical calculations were performed using GraphPad Prism 7 (GraphPad Software Inc., San Diego, CA, USA) and SPSS 25 software. (SPSS package for Windows, Release 25.; SPSS, Chicago, IL, USA). Data are presented as mean ± SEM. Statistically significant differences are denoted by *, **, and *** for *p* < 0.05, *p* < 0.01, and *p* < 0.001.

## Data Availability

Not applicable.

## Supplementary Materials

Supplementary Materials can be found at https://www.mdpi.com/1422-0067/22/4/1638/s1.

## Abbreviations

6-4PP	Pyrimidine (6-4) pyrimidone photoproduct
ABT-888	Veliparib
As_2_O_3_	Arsenic trioxide
CHO	Chinese hamster ovary cell line
CPD	Cyclobutane–pyrimidine dimer
HPRT	Hypoxanthine phosphoribosyltransferase 1
LC3	Microtubule-associated protein 1A/1B-light chain 3
mTOR	mammalian target of rapamycin
NER	Nucleotide excision repair
p53	Cellular tumor antigen p53
PARP1	Poly [ADP-ribose] polymerase 1
RSV	Resveratrol
SP	Spironolactone
XP	Xeroderma pigmentosum
UVB	Ultraviolet B radiation
