# Supplementary material for: Inhibitors of Nucleotide Excision Repair Decrease UVB-Induced Mutagenesis—An In Vitro Study
Source: Int J Mol Sci. 2021 Feb 6;22(4):1638. doi: 10.3390/ijms22041638 (PMC7915687; doi:10.3390/ijms22041638)
Supplement: Supplementary file 1 [file ijms-22-01638-s001.pdf]

Supplementary material for Fidrus et al.: Inhibitors of nucleotide excision repair decrease UVB-induced mutagenesis – in vitro study

Table S1.

**Antibody**	**Clone**	**Dilution**	**Manufacturer**
LC3I-II	D3U4C	1:100	Cell Signaling Technology, Danvers, MA, USA
mTOR	2972S	1:1000	Cell Signaling Technology
Phospho-mTOR	S2481	1:1000	Cell Signaling Technology
p53	CM 042 C	1:1000	Biocare Medical, Pacheco, CA, USA
Phospho-p53^{Ser15}	16G8	1:1000	Cell Signaling Technology
anti-PAR (10H)	sc-56198	1:500	Santa Cruz Biotechnology, Dallas, TX, USA
ß-Actin	8H10D10	1:4000	Cell Signaling Technology

Figure S1.

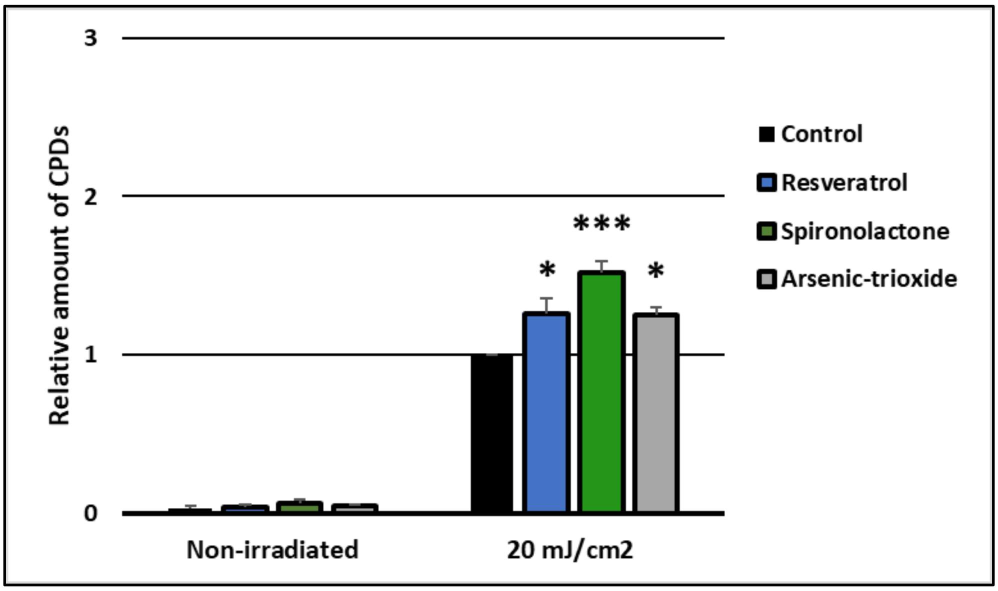

Figure S2.

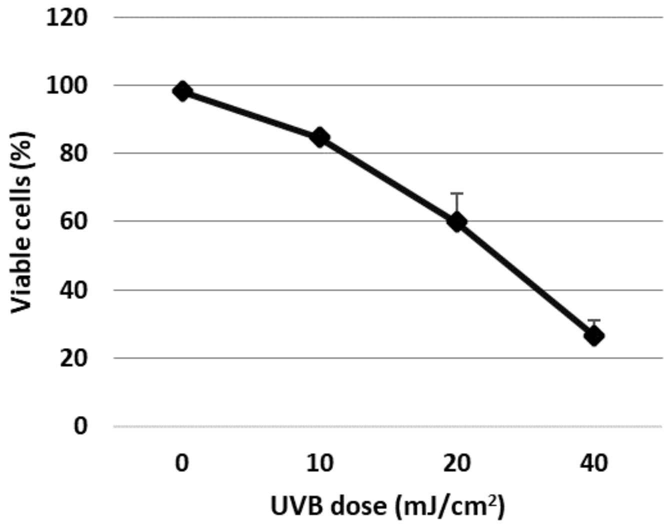

Figure S3.

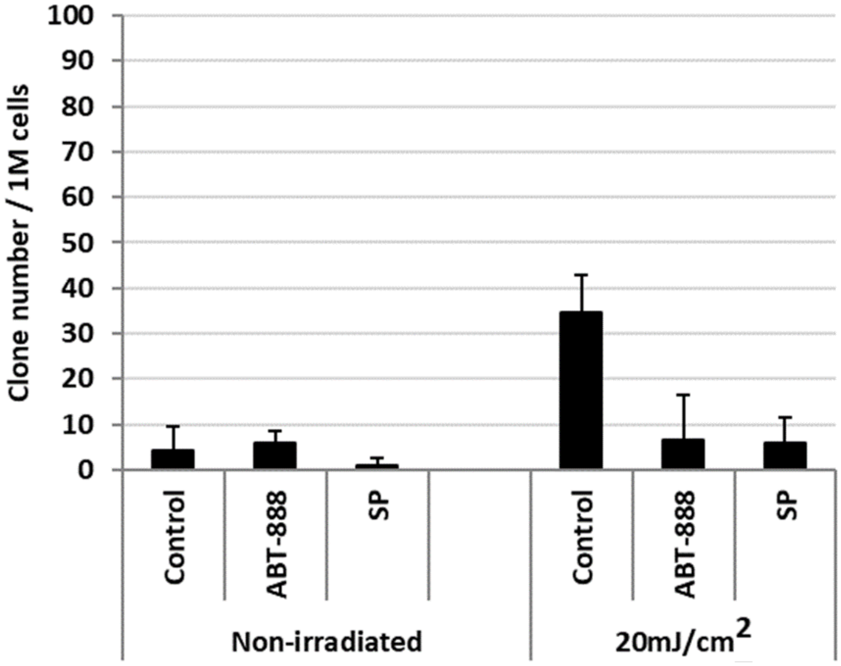

Figure S4.

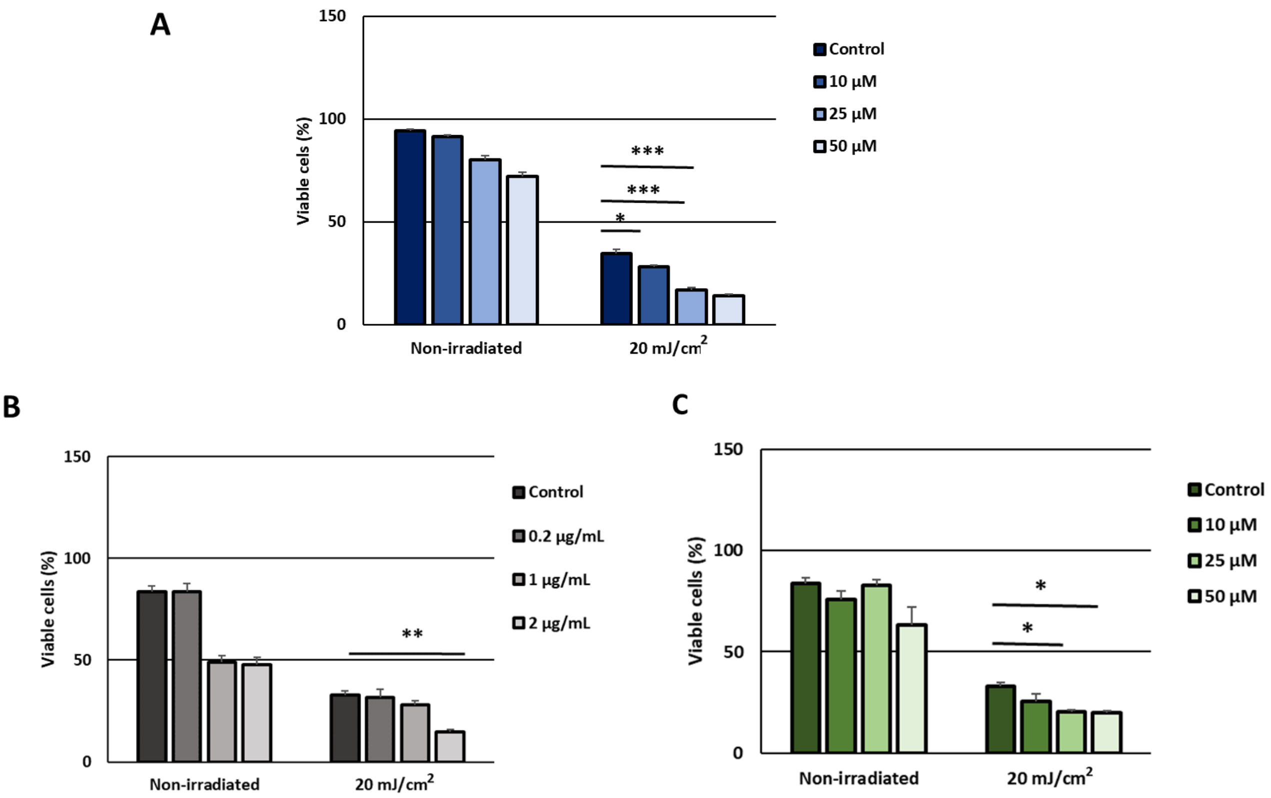

Figure S5.

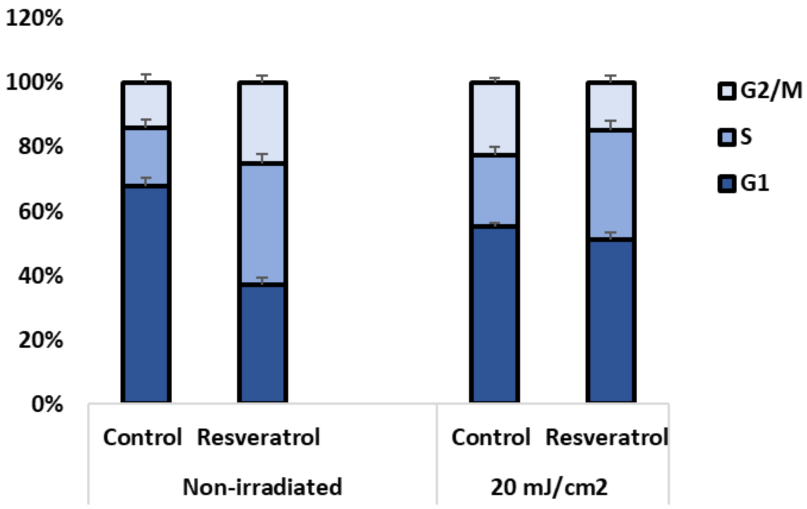

Figure S6.

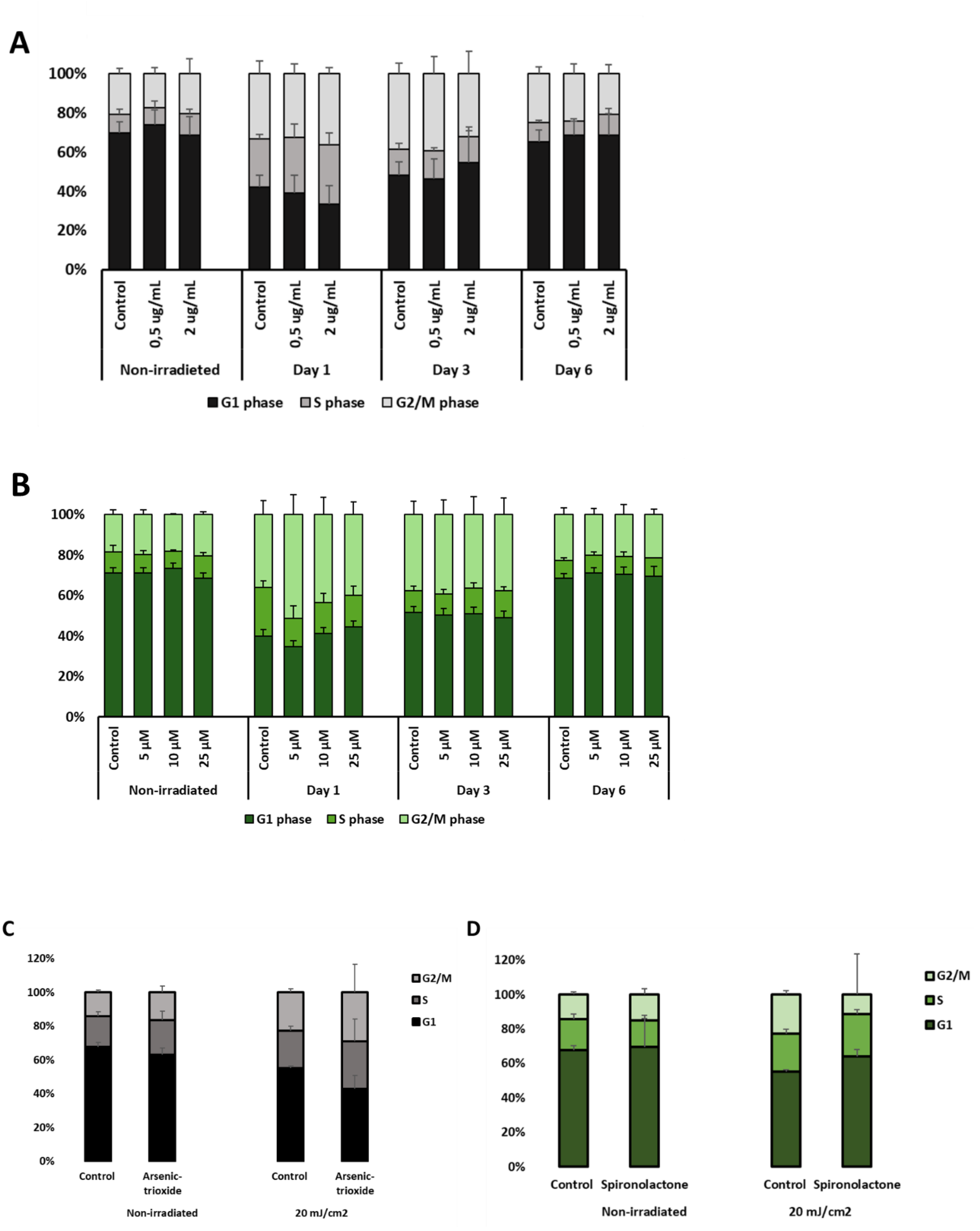
A
100%
80%
60%
40%
20%
0%
Control
0,5 ug/mL
2 ug/mL
Non-irradieted
Day 1
Day 3
Day 6
G1 phase
S phase
G2/M phase
B
100%
80%
60%
40%
20%
0%
Control
5 µM
10 µM
25 µM
Non-irradiated
Day 1
Day 3
Day 6
G1 phase
S phase
G2/M phase
C
120%
100%
80%
60%
40%
20%
0%
Control
Arsenic-trioxide
Non-irradiated
20 mJ/cm2
G2/M
S
G1
D
120%
100%
80%
60%
40%
20%
0%
Control
Spironolactone
Non-irradiated
20 mJ/cm2
G2/M
S
G1

Figure S7.

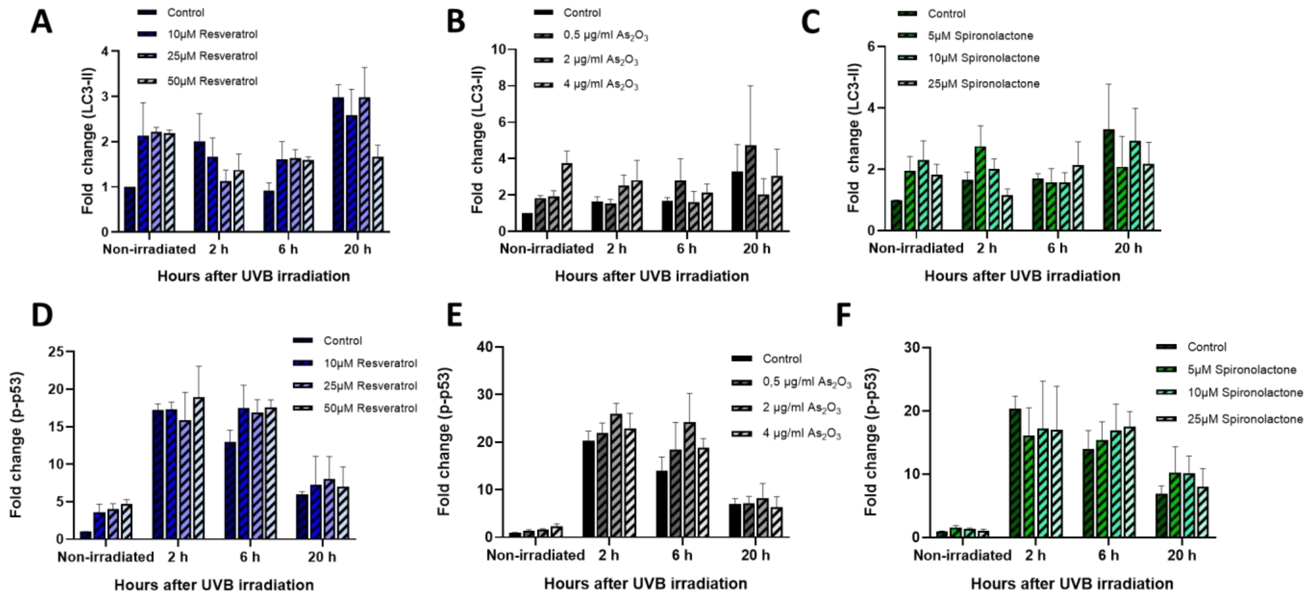

Figure S8.

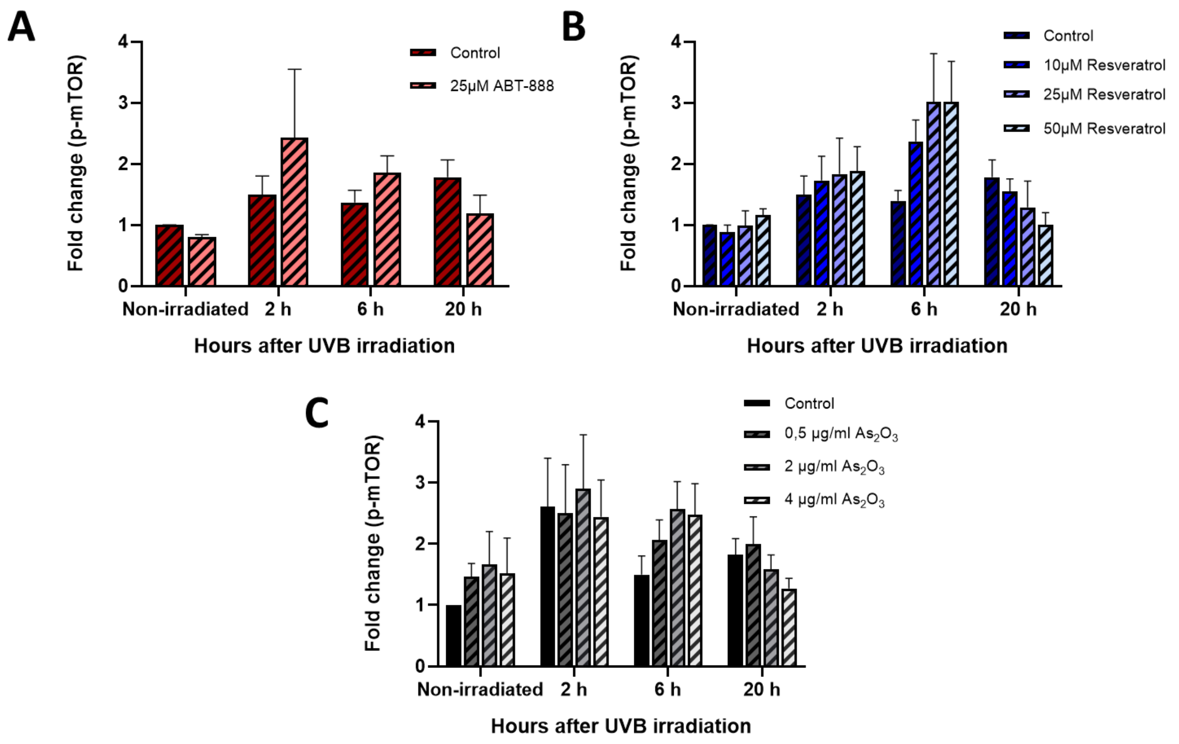

Figure S9.

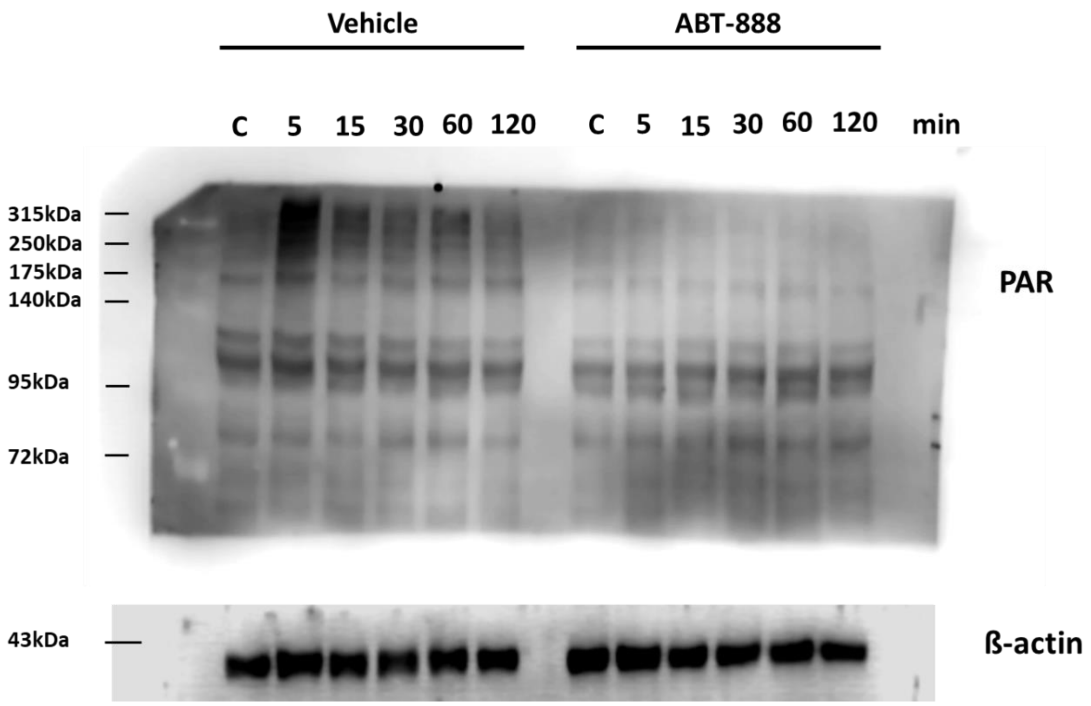
Vehicle
ABT-888
C
5
15
30
60
120
C
5
15
30
60
120
min
315kDa
250kDa
175kDa
140kDa
95kDa
72kDa
PAR
43kDa
ß-actin
